# The effect on work presenteeism of job retention vocational rehabilitation compared to a written self-help work advice pack for employed people with inflammatory arthritis: protocol for a multi-centre randomised controlled trial (the WORKWELL trial)

**DOI:** 10.1186/s12891-020-03619-1

**Published:** 2020-09-10

**Authors:** Alison Hammond, Chris Sutton, Sarah Cotterill, Sarah Woodbridge, Rachel O’Brien, Kate Radford, Denise Forshaw, Suzanne Verstappen, Cheryl Jones, Antonia Marsden, Martin Eden, Yeliz Prior, June Culley, Paula Holland, Karen Walker-Bone, Yvonne Hough, Terence W. O’Neill, Angela Ching, Jennifer Parker

**Affiliations:** 1 Centre for Health Sciences Research, University of Salford, Allerton L701, Frederick Road, Salford, Greater Manchester, M6 6PU UK; 2 Centre for Biostatistics, Division of Population Health, Health Services Research and Primary Care, University of Manchester, Manchester, UK; 3 School of Health and Wellbeing, Sheffield Hallam University, Sheffield, UK; 4 Division of Rehabilitation and Ageing, University of Nottingham, Nottingham, UK; 5 Lancashire Clinical Trials Unit, University of Central Lancashire, Brook Building, Preston, Lancashire UK; 6 Centre for Epidemiology Versus Arthritis, Centre for Musculoskeletal Research, Faculty of Biology, Medicine and Health, University of Manchester, Manchester Academic Health Science Centre, Manchester, UK; 7 NIHR Manchester Biomedical Research Centre, Manchester University NHS Foundation Trust, Manchester Academic Health Science Centre, Manchester, UK; 8 Manchester Centre for Health Economics, Division of Population Health, Health Services Research and Primary Care, University of Manchester, Manchester, UK; 9 Patient research partner, Derbyshire, UK; 10 Division of Health Research, Lancaster University, Lancaster, UK; 11 MRC Versus Arthritis Centre for Musculoskeletal Health and Work, University of Southampton, Southampton, UK; 12 Rheumatology/ Occupational Therapy, St Helens and Knowsley Teaching Hospitals NHS Foundation Trust, St Helens Hospital, St Helens, Merseyside, UK; 13 Centre for Epidemiology Versus Arthritis, Division of Musculoskeletal and Dermatological Sciences, Manchester Academic Health Science Centre, University of Manchester, Manchester, UK; 14 NIHR Manchester Biomedical Research Centre, Manchester University NHS Foundation Trust, Manchester, UK

**Keywords:** Arthritis, Vocational rehabilitation, Occupational therapy, Presenteeism, Absenteeism, Randomised controlled trial

## Abstract

**Background:**

Work problems are common in people with inflammatory arthritis. Up to 50% stop work within 10 years due to their condition and up to 67% report presenteeism (i.e. reduced work productivity), even amongst those with low disease activity. Job retention vocational rehabilitation (JRVR) may help prevent or postpone job loss and reduce presenteeism through work assessment, work-related rehabilitation and enabling job accommodations. This aims to create a better match between the person’s abilities and their job demands. The objectives of the Workwell trial are to test the overall effectiveness and cost-effectiveness of JRVR (WORKWELL) provided by additionally trained National Health Service (NHS) occupational therapists compared to a control group who receive self-help information both in addition to usual care.

**Methods:**

Based on the learning from a feasibility trial (the WORK-IA trial: ISRCTN76777720), the WORKWELL trial is a multi-centre, pragmatic, individually-randomised parallel group superiority trial, including economic evaluation, contextual factors analysis and process evaluation. Two hundred forty employed adults with rheumatoid arthritis, undifferentiated inflammatory arthritis or psoriatic arthritis (in secondary care), aged 18 years or older with work instability will be randomised to one of two groups: a self-help written work advice pack plus usual care (control intervention); or WORKWELL JRVR plus a self-help written work advice pack and usual care. WORKWELL will be delivered by occupational therapists provided with additional JRVR training from the research team. The primary outcome is presenteeism as measured using the Work Limitations Questionnaire-25. A comprehensive range of secondary outcomes of work, health, contextual factors and health resource use are included. Outcomes are measured at 6- and 12- months (with 12-months as the primary end-point). A multi-perspective within-trial cost-effectiveness analyses will also be conducted.

**Discussion:**

This trial will contribute to the evidence base for provision of JRVR to people with inflammatory arthritis. If JRVR is found to be effective in enabling people to keep working, the findings will support decision-making about provision of JRVR by rheumatology teams, therapy services and healthcare commissioners, and providing evidence of the effectiveness of JRVR and the economic impact of its implementation.

**Trial registration:**

Clinical Trials.Gov: NCT03942783. Registered 08/05/2019 (https://clinicaltrials.gov/ct2/show/NCT03942783); ISRCTN Registry: ISRCTN61762297. Registered:13/05/2019 (http://www.isrctn.com/ISRCTN61762297). Retrospectively registered.

## Background

Fifty per cent of people with rheumatoid arthritis (RA) stop working due to their condition (i.e. are work disabled) within 10 years of diagnosis [1]. Work disability seems to be reducing, possibly due to earlier aggressive drug treatment [2]. However, presenteeism (i.e. reduced work productivity) is still reported by 67%, even with low disease activity [3]. Alongside the broader economic impact of work limitations due to arthritis, the effect on individuals’ quality-of-life has also been acknowledged [4]. Job retention vocational rehabilitation (JRVR) can potentially prevent or postpone work disability and reduce presenteeism through structured work assessment, work-related rehabilitation and modifying work to suit the person’s condition and ability (termed job accommodations) and creating a better match between the person’s abilities and their job demands [5].

JRVR provision varies between countries. In the United Kingdom (UK), many with RA, early or undifferentiated inflammatory arthritis (UIA) and psoriatic arthritis (PsA) lack access to JRVR. In the UK National Health Service (NHS), work-related support in many Rheumatology services are either non-existent or patchy. Where this exists, it is usually provided in occupational therapy to only a few patients per month, often when patients are referred for other reasons. Brief work advice lasts on average 45 min, without a structured work assessment, and includes signposting to work services, providing information booklets and self-management education related to work, e.g. fatigue management, joint protection, pacing, as well as splinting) [6, 7]. In the workplace, access to occupational health is common only in larger organisations. There is some UK Government-funded support, such as the Access to Work (AtW) scheme, providing advice and grants for job accommodations [8] but there is low awareness of this amongst employees with arthritis and employers [9]. Disability Employment Advisors (working in Job Centres) usually provide advice to those already unemployed. Additionally, Fit for Work is an online resource, with an individual telephone advice service, to supplement the occupational health advice that employers may provide [10]. In Western Europe, work rehabilitation is provided by, for example: local authorities funded through the social service system (Denmark) [11]; local social insurance offices directly providing or funding services from work rehabilitation providers (Sweden) [12]; statutory pension insurance funded rehabilitation centres (Germany) [13]; and occupational physicians collaborating with health professionals, as all companies legally must have a contract with occupational health services (the Netherlands) [14]. In the United States, VR is provided by VR counsellors through federal-funded VR State Agencies [15].

There is limited evidence for effectiveness of JRVR in employed people with RA, UIA and PsA. A systematic review of randomised controlled trials (RCTs: with more than 50% participants with RA, UIA or PsA) identified two trials with positive results and one with no effects [5]. A small UK trial of occupational therapy (OT) and JRVR resulted in reduced work instability and improved self-reported ability to manage at work, at 6 months [16]. A brief JRVR intervention in the United States of America (USA) led, to reduced job loss at 4 years [17]. An RCT of a multidisciplinary JRVR programme in the Netherlands identified no changes in work outcomes, However, 40% of participants were already on long-term sick leave, suggesting the intervention may have been too late for many [14]. We systematically searched Medline, Cumulative Index of Nursing and Allied Health Literature and PubMed from 1980 to end 2018 for JRVR trials either published since the Hoving et al. [5] review or with less than a 50% sample with RA, UIA or PsA. A further trial of multidisciplinary JRVR, including an OT-led work site visit and employer liaison, also led to no changes in work outcomes in the Netherlands. Many participants had low scores on the RA-Work Instability Scale, indicating few work problems, suggesting the intervention may have been too early [18]. Two further RCTs in the USA identified positive outcomes at two-year follow-up. An OT-led workplace ergonomic intervention (38% RA) resulted in significant improvement in self-reported work impairment but not pain, job satisfaction, physical function or mood [19]. A brief JRVR intervention provided by OTs and physiotherapists (PTs) (33% IA), based on the Allaire et al. [17] trial, led to reduced job loss but not presenteeism [20, 21].

Differing economic, social security and health services between countries mean it is difficult to extrapolate whether positive results for JRVR in one country translate to another. The review indicated brief JRVR, based on those in the USA [17, 21], delivered by OTs and PTs given additional JRVR training, as part of Rheumatology health services, could be effective in the UK. Cost-effectiveness was not evaluated in these trials. Considering finite available resources, decision-makers in health and other sectors will also require information about the value-for-money that JRVR provides. Economic evaluation is a formal process which facilitates the synthesis of an intervention’s costs and outcomes to provide estimates of its cost-effectiveness.

### Feasibility study

We conducted a feasibility study, the WORK-IA trial, of JRVR for employed people with RA, UIA and PsA. In this, we: developed a JRVR training programme for OTs, effective in increasing OTs knowledge of and confidence in delivering JRVR [6]. We also modified and further developed for the UK the: Work Environment Survey for Rheumatic Conditions (WES-RC) [22, 23]; the WES-RC Manual [24]; and the brief JRVR intervention developed by Allaire et al. [17, 25]. We developed a Workwell Solutions Manual of work resources and solutions for therapists, linked to the WES-RC. We then conducted an RCT (*n* = 55), comparing a control intervention (written self-help work advice and usual care) to JRVR plus written self-help work advice and usual care. The results of this study indicated that therapists could successfully deliver JRVR, participants considered the intervention beneficial [26], and that the JRVR intervention led to greater reductions in presenteeism (measured using the WLQ-25 at 9 month follow-up) compared to the control intervention [25]. This supported the need for a definitive trial.

### Choice of comparators

As in our feasibility trial, the control group will receive a written work self-help information pack and usual care. We have chosen a self-help information pack, posted to participants, as the control intervention because patients with RA, UIA or PsA often receive little or no work advice from Rheumatology services [6, 7]. “Usual care” could include some patients receiving brief work advice from therapists but many would not. In our feasibility trial, therapists reported that it was more common for patients to receive an information booklet on work and arthritis, although many may not even receive this, if not referred to occupational therapy. We discussed with therapists what the control intervention should be: brief (i.e. about 45 min) work advice; or work information booklets. The therapists’ consensus was that, once they had received training in Workwell, they would find it more personally conflicting to provide only brief work advice to control participants, as they would now know how to provide work rehabilitation. Accordingly, their decision, supported by our patient research partners, was that they preferred not to have the control participants referred to them and that the control group should receive a self-help information pack (which is also provided to the intervention group). This pack ensures all participants receive the same high-quality written information. Additionally, this approach has been used as a control in other JRVR trials [17, 21]. The therapists in our feasibility trial agreed that the pack contained more information within the booklets than the brief advice that they might provide to employed patients. Accordingly, they considered control participants would not be disadvantaged in receiving this pack instead. Indeed, many would be advantaged, as they would not otherwise receive any work information.

For the WORKWELL trial, we have increased the content of the information pack (compared to that in the feasibility study) to include more information on the Equality Act and practical work self-help solutions and resources. If referred to occupational therapy during the WORKWELL trial for other reasons, control participants continue to receive self-management education, splinting and other interventions as part of “usual care.” Therapists were asked not to provide WORKWELL JRVR to non-trial participants during the trial. This was to avoid their normal service provision being changed by the trial as control participants could then be disadvantaged. Therapists considered this would not be feasible anyway, as their services are not normally funded to provide a full work rehabilitation service.

The intervention group will receive usual care plus the same information pack as the control group plus WORKWELL. The WORKWELL JRVR intervention is the same as developed in the feasibility trial [25]. We chose to test this intervention as systematic review identified this was effective in the USA, the most pragmatic intervention available and we have already tested it for acceptability and feasibility. We chose to deliver WORKWELL by therapists working in NHS Rheumatology out-departments because people with inflammatory arthritis are usually referred quickly to, and receive ongoing disease management from, such services, allowing for regular opportunities to identify patients’ work problems.

### Objectives

The primary objective is to assess whether there is a clinically important difference in work presenteeism (measured using the Work Limitations Questionnaire–25: WLQ-25 [27]), in people with RA, UIA or PsA receiving: WORKWELL JRVR compared to written work self-help information, in order to assess the work-related benefits to patients.

The secondary objectives are to:

i. Assess the effectiveness of WORKWELL JRVR relative to the control intervention on: work activity limitations, work instability, work productivity, absenteeism, work status, satisfaction with work advice/support received, work self-efficacy, health status and quality of life.
ii. Determine the cost-effectiveness of WORKWELL JRVR from both an NHS and employer perspective.
iii. Understand the social and structural context in which the intervention is delivered and to identify factors which may influence the quality of implementation, through conducting a process evaluation.
iv. Investigate contextual factors influencing participants’ presenteeism.

### Trial design

The WORKWELL trial is a definitive, pragmatic, multi-centre superiority randomised parallel group trial of WORKWELL JRVR compared to written work self-help advice in people with RA, UIA or PsA with work instability. Both groups will continue to receive usual care. Analysis will be on an intention-to-treat basis. Ethical approval for this study was obtained from the West Midlands – Solihull Research Ethics Committee (18/WM/0327). The study protocol was developed using the Standard Protocol Items: Recommendations for Interventional Trials (SPIRIT) guidelines [28].

## Methods

### Study setting

Study participants will be recruited from Rheumatology and Therapy departments in 22 hospitals across 18 NHS Trusts in England, Wales, and Scotland in the United Kingdom (UK). Study sites are listed in the Acknowledgements. In the UK, people with RA, UIA and PsA are predominantly treated in Rheumatology departments in secondary care and this is where therapists are most likely to be able to provide JRVR for this client group.

### Eligibility criteria

#### Inclusion criteria

- Aged ≥18 years.
- Diagnosed with RA, UIA or PsA by a Rheumatology Consultant. (UIA is defined as: persistent synovitis without any other known cause, but the person does not yet meet all the diagnostic criteria for RA [29]. Participants can have other comorbidities.
- In paid work (full or part-time, self-employed or regular contractual work) for at least 15 h per week.
- Not on sick leave **or,** if on sick leave at screening, this must be less than 4 weeks (in which case entry is deferred, and the patient is re-screened on full return to work).
- Able to read and understand English.
- Score ≥ 10 on the RA-Work Instability Scale (RA-WIS), a measure of mismatch between the person’s abilities and their job demands. A score of ≥10 is indicative of medium to high risk of work instability and need for JRVR [30, 31].
- Able to attend for WORKWELL appointments
- Able to provide informed consent.

#### Exclusion criteria

- On extended sick leave (i.e. > 4 weeks).
- Planning to retire within the next 12 months and thus unable to complete follow-up
- Planning to move out of area within the next 4 months and unable to continue to attend WORKWELL.
- Already receiving/ awaiting JRVR services from other sources e.g. Access to Work or a Vocational Rehabilitation company.
- Employed in the armed forces (which have their own JRVR service).

### Interventions

#### Control and intervention groups: treatment regimens

##### Control group

The control group will receive usual care and a written work self-help information pack.

The pack includes a letter encouraging people to work through a self-help flowchart advising on how to identify work problems and find solutions. Four work advice booklets are included, which reference further work resources [32–35].

##### WORKWELL intervention group

This group will receive usual care plus the same information pack as the control group plus WORKWELL. Within two working days of the participant being randomised, the Lancashire Clinical Trials Unit (CTU) will mail the work self-help information pack to the participant. This includes a cover letter informing them they will be contacted within the next week by a therapist who will arrange the first WORKWELL appointment as soon as possible, within a maximum of 4 weeks.

The WORKWELL intervention is a mutually-agreed, tailored, individualised programme to meet each participant’s priority work-related needs. It consists of: meetings between the participant and therapist held at mutually agreed times (early or late in the day to fit around participants’ work commitments where possible) and locations (mainly the Therapy department, but also participant’s home or workplace, if applicable) spread over two to four months. WORKWELL consists of up to four × 1-h face-to-face meetings, and a 30-min telephone review after 6 weeks to check progress. The therapist can provide treatment in shorter or longer appointments. If the participant has more complex problems and/or requires one workplace visit, a further 2 hours is allowed. The intervention starts with a structured work interview and job discussion using the UK WES-RC [23, 24]. This is an assessment of the person’s job, roles and responsibilities in relation to their condition, disease severity and activity limitations and a detailed assessment of work barriers. This is followed by mutually agreeing priority work problems, action planning, and a tailored, individualised programme including self-management at work, job accommodations, employment rights information and other strategies as relevant. The therapist uses the Work Solutions Manual to identify relevant solutions, as needed. A work site visit is conducted, if this is identified as relevant to their needs and the participant and employer agree. The participant’s responsibilities in liaising with employers are emphasised and role play included, as necessary, to enhance confidence requesting job accommodations. Therapist and participant may also liaise with UK Government-funded services e.g. Access to Work and Disability Employment Advisors, and any occupational health support available in the participant’s workplace. WORKWELL includes both direct (i.e. with the participant) and indirect JRVR (i.e. non-contact time when therapists: use resources to identify solutions for work problems; liaise with team members, other agencies and employers; complete treatment notes; and travel time to conduct work site visits). WORKWELL is normally up to 6.5 h direct and indirect JRVR in total per participant (see Supplementary Materials 1).

To improve adherence, participants are enabled to write an action plan at the end of each appointment. Progress in meeting the plan is reviewed at the beginning of the next appointment. The plan and progress are recorded in the WES-RC continuation notes. The participant is reminded about any elements still to be completed or ongoing. If a participant does not attend or respond to the therapists attempts to re-schedule the appointment (four attempts can be made by telephone and in writing), the therapist will record this and reason for non-attendance (if known) in the Treatment Record. In the feasibility trial 25/29 intervention group participants attended [25]. We therefore anticipate an 86% adherence rate with attending WORKWELL although it could be lower.

### Concomitant care

Therapists are asked not to provide work rehabilitation to control group participants whilst they are in the trial (i.e. during their one-year participation). All participants continue to receive usual care, i.e. Rheumatology clinic appointments, prescribed medication and referrals to the multidisciplinary team, as applicable, for rehabilitation. Data on participants’ use of concomitant care is collected via the baseline, six- and 12-month questionnaires.

### Criteria for discontinuing or modifying allocated interventions

The WORKWELL intervention will be delivered by the WORKWELL Therapist to meet the participant’s individual needs, selecting from the range of solutions available. WORKWELL will be discontinued only if: the treating therapist identifies a serious adverse event resulting from WORKWELL; or the participant stops attending. Serious adverse events resulting from WORKWELL are not expected due to the low risk associated with the intervention; none were identified in the feasibility study. If treatment stops, the date and reason will be recorded by the therapist. It is highly unlikely any harm will arise from receiving the work self-help information pack. Any participant discontinuing WORKWELL will remain in the trial for follow-up unless choosing to withdraw.

### Therapist training in WORKWELL JRVR

WORKWELL will be delivered by NHS therapists who specialise in rheumatology and musculoskeletal conditions. Participating therapists will attend a two-day WORKWELL training course, delivered by expert JRVR therapists (trainers) and the research team. This will include: trial background; key study procedures; how to conduct the WES-RC; case studies; practical workshops; and self-study materials. The therapists must then complete a role-play telephone WES-RC with one of the JRVR trainers, based on a case study, and write an appropriate treatment plan. Therapists will receive mentor support by telephone and e-mail from one of the JRVR trainers, including formal feedback on the WES-RC assessment and treatment plan for their second participant. All therapists are provided with the WORKWELL Solutions Manual, available in hard copy and online. The training was further developed from that delivered in our feasibility study [6]. It was emphasised that therapist should not provide WORKWELL to other patients or disseminate programme content to other therapists in order to reduce risk of contamination.

In addition, the research team will conduct site visits to ensure all Principal Investigators, research facilitator/s (i.e. nurses or other health care staff employed in the NHS to assist with recruitment into trials) and therapists involved understand how to explain the study appropriately.

### Ancillary and post-trial care

Participants are covered by indemnity for negligent harm resulting from provision of WORKWELL through the standard NHS Indemnity arrangements. The University of Salford is the sponsor for the study and has insurance to cover for non-negligent harm associated with the protocol. If the trial provides evidence of the effectiveness of WORKWELL, then control participants will be informed of this. Participants can request referral from the Rheumatology to their Occupational Therapy departments for provision of work advice. It will be the decision of participating departments whether they can provide the full WORKWELL intervention to patients or whether usual care is provided.

### Participant timeline

**Fig. 1:**
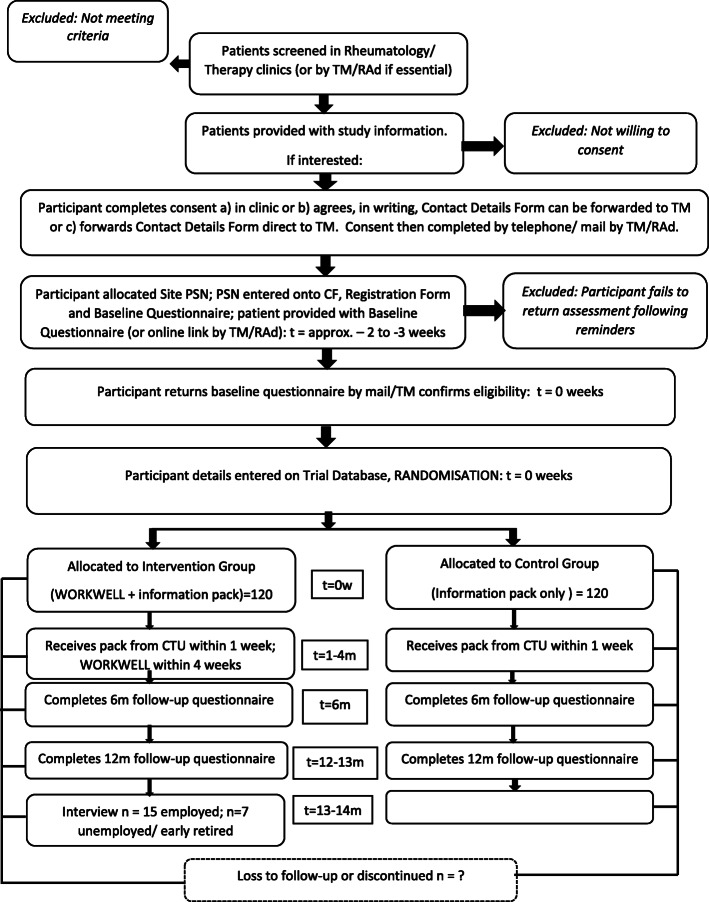
WORKWELL Trial Flowchart of participants

**Fig. 2:**
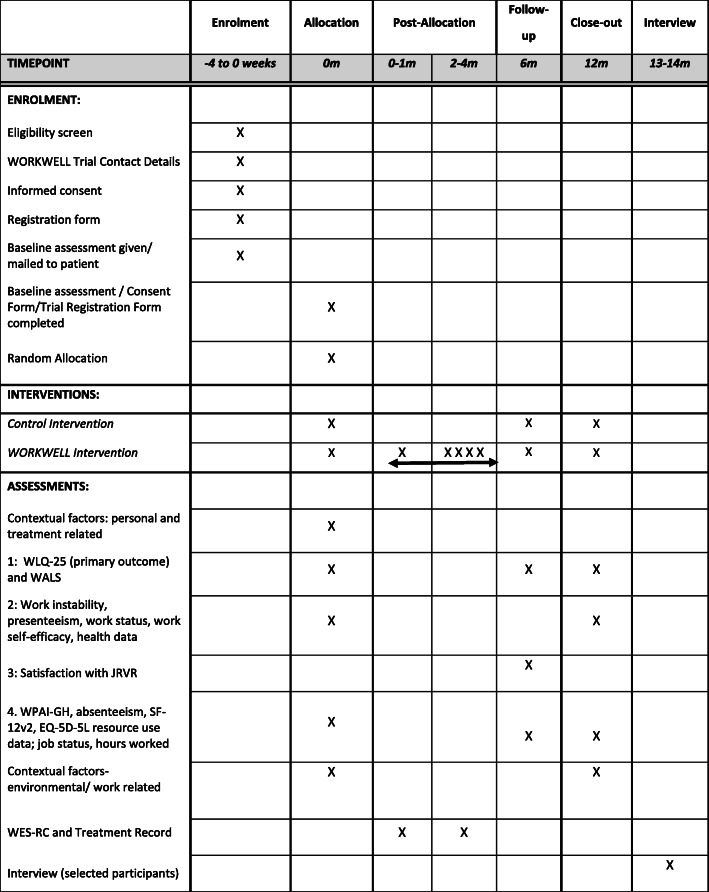
SPIRIT Flowchart: Schedule of enrolment, interventions, and assessments

After giving written, informed consent participants will complete a baseline questionnaire. Baseline is the date by which the consent form, trial registration form and questionnaire are verified as complete. Documents are normally sent that day by the co-ordinating research team to the Lancashire CTU, which randomises within two working days. After randomisation, and within two working days, all participants are mailed the work self-help information pack with a cover letter identifying their group allocation. Within 4 weeks of randomisation, a WORKWELL appointment will be arranged for those randomised to the intervention group. Participants in this group then attend a further one to three appointments (with optional work site visit) and telephone review over the next four to five months. Most participants should have completed treatment before the six-month follow-up. Participants will receive the follow-up questionnaires at six- and 12-months following the date of baseline (See Figs. 1 and 2). Following this, approximately 15 to 20 participants from the intervention group will be interviewed.

### Outcomes

The primary outcome is the Work Limitations Questionnaire-25 (WLQ-25) at 12 months [27]. Presenteeism has the greatest impact on costs for people with RA, making it the most relevant primary outcome. At the time of trial planning, the Outcome Measures in Rheumatology (OMERACT) Work Group identified the WLQ-25 and Work Activity Limitations Scale (WALS) as the most applicable presenteeism measures [36, 37]. The WLQ-25 has since been identified as having the strongest psychometric properties [38]. Accordingly, the trial sample size was calculated using the WLQ-25. Secondary outcomes include work instability, additional presenteeism measures, work status, absenteeism and work self-efficacy, as the focus of the intervention is affecting the work environment and the person’ ability to meet their job demands. Additionally, we are including: health outcomes to investigate whether enhancing work ability impacts on health status; contextual factors which may influence work ability; and resource use and costs to measure cost-effectiveness of WORKWELL (Table 1).

**Table 1:** Outcome measures in the WORKWELL trial

Data collection	Measurement Method	Details	0-Months	6-Months	12-Months
**Contextual factors: personal (demographic; disease status)**					
Age	Date of birth		✓		
Gender			✓		
Ethnic group			✓		
Marital status			✓		
Living status		Alone; or with family/ significant others	✓		
Educational status		Highest qualification	✓		
Time since RA/ IA/ PsA symptom onset			✓		
Time since RA/ IA/ PsA diagnosis			✓		
Comorbidities	Rheumatic Disease Comorbidity Index [39]	Presence/absence of 11 comorbidities	✓		
Job Descriptors		a) number of hours worked/week; b) contract type/ self-employment; c) length of time in current main job (0 m only).	✓	✓	✓
Income status		Sole income earner in household	✓		
**Primary Outcome measure:**					
Presenteeism	The Work Limitations Questionnaire^a^ (WLQ-25) [27]	25 items: percentage time (in last 2/52) limited in: physical work demands, time demands, mental-interpersonal demands and output demands (0–4 scale of 0 to 100%).	✓	✓	✓
**Secondary outcome measures**					
Presenteeism	Work Activities Limitations Scale^a^ (WALS) [40]	12 items: degree of difficulty in work activities (0 = no difficulty; 3 = unable to do)	✓	✓	✓
	Combined WLQ-25 WALS^a^ [41]	Combination of above two measures	✓	✓	✓
	WLQ-25 [27]	4 subscales calculated from relevant items in the WLQ-25 questionnaire	✓	✓	✓
Absenteeism and Presenteeism	Work Productivity and Activity Impairment Questionnaire General Health V2.0 (WPAI-GH) [42]	Last 7 days: time off on sick leave/any other reason; hours worked; health problems effect on work productivity and ability to do other daily activities.	✓	✓	✓
Work status	Work Transitions Index [43]	Whether employed; in full- or part-time work	✓	✓	✓
Work instability	Work instability scale (RA-WIS) [30]	23 true/false items: degree mismatch between functional abilities and workplace demands	✓		✓
Work self-efficacy		0–10-point numeric rating scales (NRS) 0 = not at all to 10 = very. 3 work self-efficacy items (confidence in working, managing health condition at work and work not making condition worse (0–10 NRS)	✓		✓
**Health Outcome measures**					
Health status	SF-12v2 Health Survey [44]	12 items, assessed over the last four weeks, scored as physical and mental health sub-scales	✓	✓	✓
Disease impact	Rheumatoid Arthritis Impact of Disease (RAID) [45]	Composite score from: 7domains of impact of RA: coping, emotional wellbeing, fatigue, physical function, sleep, global assessment and pain	✓		✓
Disease activity	RA Disease Activity Index-5 (RADAI-5) [46]	Self- reported disease severity in last 6 months; current joint tenderness and swelling; pain, general health; hand joint stiffness duration	✓		✓
Hand/ wrist pain		In the last week during moderate activity (e.g. cooking, housework): 0–10 NRS	✓		✓
**Exploratory outcome measures**					
Number of hours worked		On average, number of hours contracted to work and number of hours usually work	✓	✓	✓
Change in occupation or hours		Any change in occupation or number of hours worked with reason for change		✓	✓
Work self-efficacy		Confidence, motivation and importance of continuing to work over next year, scored 0–10	✓		✓
Work advice received		Satisfaction with work advice received (0–10 NRS) and what other work advice they received from where		✓	
**Economic Evaluation: Use of Resources**					
Health related Quality of Life	EuroQol Five Dimensions questionnaire (EQ-5D-5L) [47]	5-items Scale (Mobility; Self-care; Usual activities; Pain/Discomfort; Anxiety/Depression; plus 0–100 scale	✓	✓	✓
Use of NHS and Social Care, Local Authority and Private Health Services	Your use of hospital in-patient services	a) Any planned hospital overnight stays in the last 6 months b) If yes, department, and number nights	✓	✓	✓
	Your use of hospital out-patient appointments	a) Any planned hospital outpatient appointments lasting 4 h or less in the last 6 months b) If yes, department and number of appointments	✓	✓	✓
	Your use of day hospital appointments	a) Any day or hospital outpatient lasting more than 4 h but not overnight during the last 6 months b) If yes, department, and number of appointments	✓	✓	✓
	Your use of accident and emergency services	a) Any A&E attendance in the last 6 months b) If yes, number of visits not leading to hospital admission c) number of visits admitted into a hospital as an in-patient from A&E d) reason for admission	✓	✓	✓
	Your use of primary and community-based health services; Local Authority Social Services; private health services	a) Use of primary care services / Local Authority Social Services (e.g. GP, Practice Nurse, District Nurse, Counsellor, social worker, occupational therapist) in the last 6 months b) If yes, number of visits to each	✓	✓	✓
	Medication	Current medication for RA/IA/PsA; date medication started	✓	✓	✓
Workplace changes	Changes to workplace environment	Changes made by employer (e.g. specialised equipment).	✓	✓	✓
**Contextual factors: environmental factors**					
Nature of work	Job skill level 1–4 (UK Standard Occupational Classification (SOC2010)) [48].	Main job title and industry working in	✓		
	Job responsibilities	e.g. overtime, variable hours; and need to travel for business	✓		
	Work demands	Two items: physical and mental work demands	✓		✓
	Control over work and flexibility [49]	Four items: control over work schedule, job flexibility, ability to postpone work tasks and help from colleagues with work	✓		✓
	Work productivity	One item: self-reported work productivity in the last 6 months	✓	✓	✓
Work Transitions	The Work Transitions Index (WTI)^a^ [43].	Includes a) employment status; b) 14 items assessing: job disruptions due to arthritis c) sickness absence due to arthritis or other conditions in the last 6 months; d) change in occupation or working hours in the last 6 months.	✓	✓	✓
Workplace Support	The Perceived Workplace Support Scale (PWSS) [50]	a 19-item questionnaire, of perceived managerial, co-worker and organisational support (REF)	✓		✓
	Work support services	Access to occupation health and work support services	✓		✓
Work Policies and Accommodations	The Workplace Accommodations, Benefits, Policies and Practices Scale (WABPPS) [51, 52]	17 items: job accommodations, policies and workplace practices available, use of and helpfulness	✓		✓
Organization Polices	Organization size	Numbers of employees in company	✓		
**Contextual Factors: Personal**					
Personal appraisal	Job strain [53]	One item: stress currently experienced at work	✓		✓
	Work motivation/ importance/self-efficacy	3 items: motivation, importance and confidence to continue working (0–10 NRS) (0–10 NRS)			
	Disclosure [50]	Disclosure about condition to employer /line manager	✓		✓
	Job satisfaction	Satisfaction with job: 0–10 NRS	✓		✓
	Future job expectations	Two items: concerns about health affecting ability to work in future; and likelihood of leaving job in the next year due to arthritis	✓		✓
Work-Life Balance	Work-life balance [54].	Four items: perceived work-life balance taken from the Work, Health Life Balance Perceptions Scale	✓		✓

Notes: ^a^ the WLQ-25 and WALS will be collected within the Combined WLQ-25 WALS dual scored measure [41]

Each month, employment status and absenteeism will be collected by the Lancashire Clinical Trials Unit via e-mail or telephone

### Data collection

The baseline questionnaire includes: demographic factors i.e. age, gender, living situation, ethnicity, educational qualifications; employment factors, i.e. employment status, main job title; hours worked, job features (e.g. working overtime, shift work), organisation size, work-related support services accessed; and condition specific factors, i.e. symptom duration, time since diagnosis. For each of the following outcomes, the metric will be the final value, and aggregated as means. The baseline, six- and 12-month follow-up questionnaires include (unless otherwise stated):

#### Work- related outcome measures

i) The Work Limitations Questionnaire (WLQ-25): a 25-item reliable, valid measure of presenteeism, indicating the amount of time, in the last 2 weeks, a person was limited in: physical work demands, time demands, mental-interpersonal demands and output demands. The summed score (i.e. of the four sub-scales) will form the primary outcome measure [27].

Secondary outcomes include:

ii) **Presenteeism**:

- WLQ-25 sub-scales: the four subscales measuring physical work demands, time demands, mental interpersonal demands and output demands from specific items in the WLQ-25 questionnaire [27].
- The Work Activities Limitations Scale (WALS): a reliable, valid measure including 12 items assessing degree of difficulty performing work activities (0 = no difficulty; 3 = unable to do [40].

The WLQ-25 and WALS will be collected using the combined WLQ-25 and WALS, from which each can be calculated, as well as a dual-key scored outcome [41]. Subsequent to the trial starting, this dual-key scored outcome is now supported by the OMERACT Work Productivity Group as the current best available measure of presenteeism, and a better predictor of work cessation than the original WLQ-25 [41, 55].

- The Work Productivity and Activity Impairment Questionnaire General Health V2.0 (WPAI:GH): a reliable, valid measure of extent of absenteeism, presenteeism, and impairment in daily activities attributable to general health [42].

iii) Work status: whether in full- or part-time work at six and 12 months, collected using the Work Transitions Index [43];
iv) Absenteeism**:** each month, the number of days sickness absence attributable to arthritis or other causes. This is collected by text, e-mail or telephone.
v) RA-Work Instability Scale (RA-WIS): at 0 and 12 months: reliable, valid measure including 23 true/false items measuring mismatch between functional abilities and job demands [30]
vi) Work Self-efficacy: at 0 and 12 months: three 0–10 numeric rating scales (NRS) asking about confidence in: ability to work; ability to manage their health condition at work; and working will not make their health worse. Additionally, three 0–10 NRS will be used to measure: motivation; importance and confidence to continue working over the next year.

#### Health outcome measures

i) SF-12v2 Health Survey: a reliable, valid measure including 12 items scored as physical and mental health sub-scales [44].
ii) Rheumatoid Arthritis Impact of Disease scale (RAID): a valid, reliable measure assessing seven domains of impact of RA: coping, emotional wellbeing, fatigue, physical function, sleep, global assessment and pain [45]. This will be measured at 0 and 12 months.
iii) RA Disease Activity Index-5 (RADAI-5): identifying disease activity categories based on perceived disease severity in last 6 months, and current joint tenderness and swelling, pain, general health and hand joint stiffness [46]. This will be measured at 0 and 12 months.
iv) Hand/ wrist pain: pain in the last week during moderate activity (0–10 NRS), measured at 0 and 12 months.

#### Health economic outcome measures

i) Health related quality of life**:** measured using the five-level version of the EuroQol (EQ-5D-5L) [47]. The EQ-5D is recommended by the National Institute of Health and Clinical Excellence (NICE) for use in the economic evaluation of healthcare interventions.
ii) Health and work resource use questionnaire**:** a bespoke tool for determining the extent of trial participants’ primary, secondary and private health care use over the preceding six-month period. The tool also prompts individuals to recall their use of medications and any changes made in the workplace relating to their condition.

We will also collect information on: exploratory outcomes, which we will compare across randomised groups, but will not be considered as formal secondary outcomes and work-related contextual factors to explore their potential impact on outcomes (listed in Table 1).

### Process evaluation

An embedded process evaluation informed by Normalisation Process Theory will explore how WORKWELL is understood, valued, adopted into practice and monitored by those involved [56]. It will include interviews with participants, employers, therapists and their line managers, as well as analysis of therapists’ WES-RC assessment and trial treatment records. Additionally, we will check treatment fidelity through analysis, for each site, of at least one audio-recording of a participant’s initial assessment and analysis of that participant’s WES-RC and trial treatment record. This will investigate whether: the WES-RC assessment was conducted correctly; and an applicable treatment plan developed and delivered, in relation to the recorded problems identified and recorded. A separate process evaluation protocol will be prepared.

### Sample size

This was calculated using data from the feasibility study. The minimal clinically important difference for the WLQ-25 is estimated as 13 points in the summed score [57]. The standard deviation (SD), pooled across the intervention and control groups, was 20.64 in the feasibility trial. As the SD might be an under-estimate, the 80% upper one-sided confidence limit of the estimated SD, i.e. 23.02, was used. Although the WORKWELL trial is individually, rather than cluster randomised, we are concerned that the effect of the WORKWELL intervention may vary by therapist (as there is more than one therapist at 11 of the 22 hospitals in the 18 sites). This could lead to a clustering effect on outcome. No such effect is expected in the control intervention. We therefore performed the sample size calculation using the *clsampsi* Stata command [58]. As we do not expect the clustering effect to be large, the intra-class coefficient (ICC) for the WORKWELL intervention arm = 0.05 and control arm = 0. To identify a 13-point difference, SD = 23.02, *p* = 0.05 and 90% power, 90 participants are required in each group. Allowing for a 25% attrition rate at 12 months (i.e. non-completion of the WLQ-25 at 12 months due to no longer working or on long-term sick leave; or non-return of the 12-month questionnaire) we intend to recruit and randomise 240 participants in equal numbers to the two groups. We anticipate we may need to obtain consent from up to 300 patients to achieve 240 participants at randomisation (as some will not complete and return their baseline questionnaire, and some may become ineligible prior to randomisation) and 180 participants with 12-month outcome data.

### Recruitment

At each participating site a Principal Investigator (PI) (senior therapist/consultant rheumatologist) will be appointed, to be responsible for identification, recruitment, consent and provision of baseline questionnaires, along with adherence to the study and treatment protocols, following Good Clinical Practice Guidelines (GCP).

**Fig. 3:**
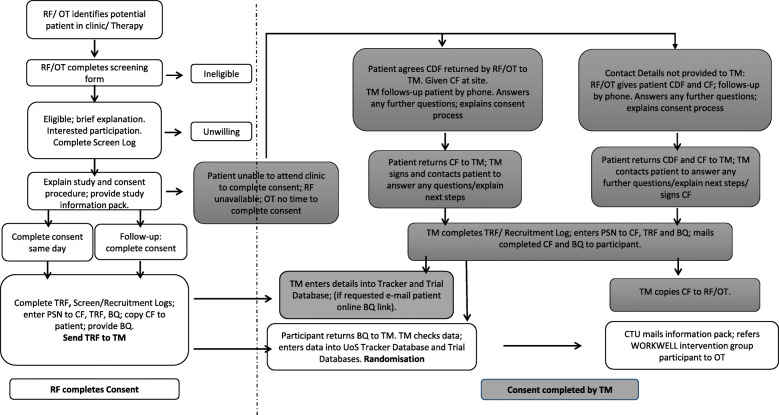
Recruitment flowchart: participants identified in rheumatology clinic/occupational therapy

Members of the health care team and therapists at participating sites will identify adult patients with RA, UIA or PsA during the patient’s Rheumatology or rehabilitation appointment. Either a research facilitator or therapist will then screen (in person or by telephone) patients for eligibility using the WORKWELL Trial Eligibility Screening Form. (See Fig. 3 for recruitment procedure). All eligible patients will be provided with a study explanation and information pack.

**Fig. 4:**
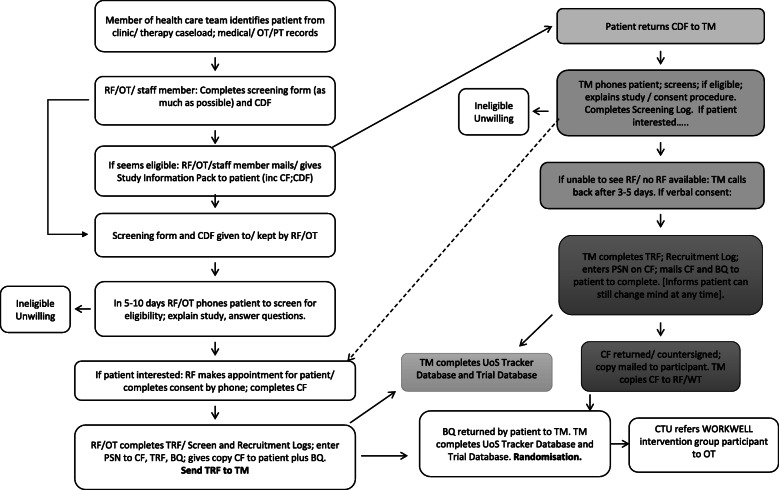
Recruitment flowchart: participants identified from records/ clinic and not seen in person

If a site is encountering difficulty identifying enough numbers during clinic appointments, then potential participants will be identified from medical or therapy records by members of the health care team. The patient will then be mailed a study information pack by the research facilitator or therapist. On return of a WORKWELL Contact Details Form from the patient indicating interest in the study, the research facilitator or therapist will then complete recruitment and consent procedures by telephone and mail. (See Fig. 4 for recruitment procedure.)

In some cases, a research facilitator or therapist may be unable to either screen, explain the study, and/or take consent in person or by telephone with a patient (e.g. the patient cannot stay in or return to clinic as they need to be in work; NHS staff are unable to telephone the patient in the evening; NHS staff have insufficient time/ are unavailable to complete the activities). In these cases, following the patient’s written consent for their contact details to be forwarded to the co-ordinating centre (at the University of Salford), a member of the research team will complete these activities.

Consent is completed by the research facilitator, therapist or member of the co-ordinating research team. Participants are also asked to consent to being contacted in future to ask about taking part in a longer-term follow-up for this study, and other associated studies. The participant information sheet and consent form can be seen in the Supplementary Materials. Following consent, participants will be provided with a baseline questionnaire to complete at home and return in a Freepost envelope to the trial manager. On receipt of the completed questionnaire, the trial manager will enter their details into the trial spreadsheet, securely e-mail scanned copies of the trial registration form, consent form and baseline questionnaire to the Lancashire CTU and request that the unblinded CTU staff perform the randomisation.

### Assignment of intervention

#### Allocation and sequence generation

Participants will be randomly assigned to either the WORKWELL intervention or control with a 1:1 allocation as per a computer-generated randomisation schedule. Participants will be stratified by their skill level group (two strata: Stratum A - Level 1 (elementary occupations) or 2 (Administrative, caring, leisure, sales, customer service; process, plant and machinery operatives); Stratum B - Level 3 (Associated professional and technical/ skilled trades) or 4 (Professional and managerial) [48], using permutated blocks of random sizes. Stratified randomisation ensures equal distribution of job skill levels across the two groups.

#### Concealment mechanism

Participants will be randomised using ‘Sealed Envelope,’ a secure, online, central randomisation service [59]. The block sizes or schedule will not be disclosed to the therapists, research facilitators or research co-ordinating team to ensure concealment. Randomisation will occur only after the trial manager and CTU confirm a participant’s eligibility, consent, trial registration and that they have completed the baseline questionnaire.

#### Implementation

Within two working days of notification by the trial manager, unblinded CTU staff will perform the randomisation and securely e-mail a referral for either intervention or control to the relevant site. Within two working days of the participant being randomised, the CTU will mail the work self-help information pack to the participant. For those randomised to WORKWELL, the treating occupational therapist will contact the participant by telephone, text or e-mail within five working days of referral to make a mutually convenient appointment to commence WORKWELL as soon as possible and within 4 weeks of referral.

### Blinding

Due to the nature of the intervention it will not be possible for therapists to be blinded to group allocation. Therapists are informed not to provide the WORKWELL intervention to any other patients. None of the participating sites normally conduct full work assessments and detailed work provision. To reduce the risk of contamination of the control group (i.e. therapists providing control participants with JRVR), WORKWELL therapists will be informed which participants are in the control group. They will enter the participant’s details onto the Control Participant Log within the Trial Therapy site file. Therapists are requested to check with patients whether they are in the WORKWELL trial and check this Log before providing any brief work advice to patients. Participants cannot be blinded to the intervention they receive. However, we will present the study in the Participant Information Sheet as a trial comparing two methods of providing work advice, without any suggestion that one is preferable. The study explanation includes the conflicting evidence from previous trials and that full JRVR is rare in Rheumatology services. Thus, patients will be informed that, in either arm, they will receive more information about managing work problems than normally available. The trial manager and co-ordinating research team are not blinded to group allocation.

Data co-ordination and data entry staff at the CTU responsible for baseline and follow-up questionnaire management and contacting participants to obtain any missing data at follow-ups, will be blinded to group allocation. The CTU trial manager, data manager and information systems team will be unblinded. The Trial Statistician will initially conduct the analyses blinded to group allocation, ignoring the therapist clustering effects as these cannot be accounted for without knowing group allocation. After the results under blinding have been shared, the Trial Statistician will become unblinded and the analyses will be re-run accounting for therapist effects. Adjusting for therapist effects will only impact the precision of estimates, rather than their magnitude. The health economist will be not be blinded as the costs of WORKWELL will be included in the analysis.

### Emergency unblinding

Not applicable as serious adverse events are not known to occur in clinical practice.

### Data collection methods

Outcomes will be collected via self-reported questionnaires at baseline (i.e. prior to randomisation) and follow-up after six and 12 months. At six-month follow-up, participants will normally have completed their intervention (which normally extends between three to five months and should be started within 4 weeks of randomisation) and have been progressively implementing job accommodations and self-management at work. Data collection is via postal questionnaire with an on-line option available at six and 12 months, provided via e-mail with a weblink (using Research Electronic Data Capture (REDCap) [60]. The trial manager (baseline) and CTU (six and 12 months) will monitor return of questionnaires.

• **At 2 weeks** after questionnaire provision/mailing, if the questionnaire is not yet returned, the trial manager [baseline questionnaire] or CTU [six- or 12-month questionnaire] will text/e-mail/ telephone (as applicable) to remind the participant to return their questionnaire.

• **At 3 weeks** after questionnaire provision/mailing, if the questionnaire is not yet returned, the trial manager [baseline questionnaire] or CTU [six- or 12-month questionnaire] will mail a reminder letter and a further copy of the relevant questionnaire (with Freepost envelope).

• **If the six- or 12-month questionnaire are not returned by seven and 13 months, respectively,** the CTU will then telephone the participant to obtain a minimal data set. This will include at least the primary outcome measure (in the form of the WLQ-25 – WALS combined measure). The following key outcomes will also be collected if possible: job status (WTI part 1, if currently on sick leave, and date of stopping/changing job, if applicable; the WPAI, EQ-5D-5L; thereafter, the Work Transitions Index (WTI) parts 2,3 and 4, health resource use, work-related questions, and the SF-12. At 12 months, the RA-WIS will also be collected. If the CTU are unable to obtain this data, this will be recorded in the Trial Database and data entered as missing.

### Data management and confidentiality

#### Data validation

Data management personnel will validate each questionnaire prior to data entry, with self-evident errors corrected, and recorded. The trial manager or CTU trial administrators will contact the participant for clarification if required.

#### Data entry and coding

Data received as hard copy will be entered into the trial database by the CTU data entry personnel. Data entered online will be automatically entered into the trial database. For every 50 questionnaires entered, data entry will be verified by batch sampling a minimum of 10% of case report forms and verifying against the database entries. A record of errors will be recorded together with actions and resolutions. The type and number of errors found will inform the decision about increasing the verification scope and frequency, up to full data set verification. A Data Management Plan will be approved by the Trial Management Group (TMG) prior to implementation.

#### Confidentiality and data storage

Information about study participants will be kept confidential and managed according to the requirements of the Data Protection Act, NHS Caldicott Guardian, the Research Governance Framework for Health & Social Care, Ethics Committee Approval and University of Salford Research Governance Procedures. All study-related information will be stored securely at the University of Salford and Lancashire CTU. Data on central servers at either location is stored in password-protected restricted access folders only accessible to the relevant research teams at those sites. The REDCap secure web application for online questionnaire collection meets the requirements of internationally recognised International Council for Harmonisation of Technical Requirements for Pharmaceuticals for Human Use (ICH) Good Clinical Practice, Federal Drug Agency 21 Code of Federal Regulations Part 11 and the European Union Clinical Trials Directive. Data hosting storage exceeds International Organisation for Standardisation 27,001 Information Security Management requirements. All data collection, process, and administrative forms will be identified by a coded Patient Screening Number (PSN) or Participant Identification Number (PIN). Patient-identifiable data will be securely stored separately from data identified by PSN or PIN. Original baseline questionnaires, participant documents, treatment and interview audio-recordings and transcriptions will be securely stored at the University of Salford. Any hard copy six- and 12-month questionnaires will be stored at the Lancashire CTU until transferred to the University of Salford at the end of the study. Interview recordings will be deleted following transcription and analysis. Original data and records will be archived for 6 years at the Centre for Health Sciences Research, University of Salford.

#### Data transfer from the University of Salford to CTU

The trial manager at the University of Salford will ensure copies of all completed baseline questionnaires are provided, in a timely and secure manner (i.e. scanned and sent by e-mail using encrypted PDF), to the CTU for data entry.

#### Data access

The CTU Principal Clinical Trials Manager and Data Manager will oversee intra-study data sharing, with input from the Trial Statistician. To ensure confidentiality, data dispersed to the Supervising and Trial Statisticians, health economist, and contextual factors team will be blinded of any identifying participant information. Following analysis, the Chief Investigator will receive a copy of the final cleaned raw and final datasets and Stata code for the coding of data and analysis. The members of the Process Evaluation team will also have access to the pseudo-anonymised interview data, interview and treatment audio-recordings, WORKWELL WES-RCs and Treatment Record Forms.

### Statistical methods

Primary effectiveness analyses will follow a pre-specified Statistical Analysis Plan (approved by the Trial Steering Committee (TSC) and available on request) and will include the intention to treat population. Data will be summarised using means, medians or proportions as applicable. The primary analysis will use mixed effects linear regression to estimate the effect of group allocation on WLQ-25 summed index scores, controlling for the stratification variable in the randomisation process (skill level group) and the baseline value of the WLQ-25 (both fixed effects) and therapist (random effect); the six- month and 12-month outcomes will be analysed in a single model with an interaction term between time point (six or 12 months) and randomised group to determine the treatment effect at both time points separately. Secondary effectiveness analysis will repeat the primary analysis method for all other outcomes, using appropriate generalised linear mixed modelling methods (i.e. mixed effects linear, logistic or ordinal logistic regression) including six- and 12-month outcomes as available, controlling for the stratification variable, baseline value of the specific measure and therapist effect. The interaction between time point and randomised group will not be included for outcomes measured only at 12 months. Sensitivity analyses will include the analysis of the primary outcome measure excluding anyone allocated to the intervention who did not attend WORKWELL and anyone allocated to control but did receive WORKWELL (the per protocol population). We do not anticipate undertaking sensitivity analyses on the basis of missing data unless there is a great deal and/or large imbalance in missingness between the two trial groups.

We will also explore the effects of contextual effects on outcomes via sub-group analyses. This will be an exploratory analysis for hypothesis generation. Each sub-group analysis will be specified in advance of starting analysis, with a theoretical justification for its inclusion. Analysis will be based on tests of interaction between group and contextual factors, as part of regression models. Interpretation will not over-emphasise any statistical or non-statistical significance at the traditional 5% level given the likely lack of power, multiple sub-group investigations and expectation that any sub-group effects will not be substantial. The contextual factors analysis will be fully described in the Statistical Analysis Plan.

#### Economic evaluation

A cost-effectiveness analysis (CEA) approach will be used in the economic evaluation. The base case analysis will be conducted in line with the NICE Reference Case [61]. The costs and outcomes of WORKWELL will be compared to those associated with the provision of usual care plus the information pack. Additional economic analyses will consider alternative outcomes measures and perspectives (see *E*co*nomic analysis* section below).

#### Costs

Costs to be included in the analysis will reflect those incurred by the NHS in terms of providing care in the control arm and the intervention arm along with any use of NHS services over the one-year time horizon of the CEA. Unit costs, identified from relevant sources (e.g. Department of Health reference costs), will be attached to the collated resource use data to provide overall costs for each arm of the study.

#### Outcomes

In the base case analysis, Quality-Adjusted Life Years (QALYs) will be generated for each arm of the trial using the EQ-5D-5L scores mapped onto EQ-5D-3L utility values as recommended by NICE [62]. The SF-12v2 health status measure will be used to generate QALYs in an additional CEA. Further analyses will employ the WLQ-25 and the WPAI as outcome measures for CEAs (see Economic analysis section below).

#### Economic analysis

Net costs and outcomes for each arm of the trial, adjusted for baseline characteristics, will be estimated in regression models. Using an NHS perspective in the base case CEA, an incremental cost-effectiveness ratio (ICER) will be calculated to reflect the cost per unit difference in QALYs associated with WORKWELL compared to usual care plus information pack provision. Bootstrapping – where multiple ICERs are generated through the resampling of trial data – will be used to characterise uncertainty in the combined cost and outcomes data. Cost effectiveness acceptability curves, populated with bootstrapped data, will be used to illustrate the probability that WORKWELL is cost-effective given the NICE-recommended threshold of acceptability [61].

Additional scenario analyses will comprise:

- a CEA conducted in line with the base case analysis but using the SF-12 to generate QALYs; previous work has indicated that this tool is particularly appropriate for the economic evaluation of work-based interventions
- a CEA in which the ICER represents the cost to employers for reduced presenteeism as measured by the WLQ-25
- a CEA in which the ICER represents the cost to employers for reduced presenteeism as measured by the WPAI
- a CEA in which the ICER represents the cost to employers for QALY gains and where levels of presenteeism, as measured by the WLQ-25, are translated into costs using the human capital approach

### Data monitoring

The trial will not have a separate Data Monitoring Committee, as the safety risks associated with this study are very low. Consequently, there are no stopping rules for safety. The trial will be overseen by a TSC, which will perform this function.

### Harms

There is minimal risk associated with the intervention. Any adverse event considered by the occupational therapist to be resulting from participation in the trial will be recorded by the occupational therapist on the WORKWELL Treatment Record Form during treatment sessions. This is completed by the six-week review and a copy returned to the CTU Data Manager. If a participant notifies the therapist of any other adverse events after treatment is completed, then the therapist will e-mail the CTU Data Manager, who will record this additionally on the participant’s WORKWELL Treatment Record form.

### Auditing

The Trial Manager or Chief Investigator will conduct at least one on-site monitoring visit per year over the course of the study to all clinical sites to educate, support and solve problems. A site file checklist will be used at the visit to ensure the site file is up to date. The trial manager or chief investigator research team will complete the checklist and arrange for any missing documents to be added. There will also be remote monitoring, by the CTU and Trial Manager, of quality of source documentation, including consent form completion, adverse event reporting, and deviations from the protocol.

The Chief Investigator will permit study-related monitoring, audits and inspections by the Ethics Committee, lead Research and Development department, the University and any NHS Trust Research Governance Managers requiring this. The study will be monitored in accordance with NHS and University Research Governance procedures. The Chief Investigator will ensure that any regulatory authority is given access to all study-related documents and study related facilities. Principal investigators will be asked to audit their site files at the beginning and end of the study using a checklist provided by the University of Salford co-ordinating research centre and to allow study-related monitoring, audits and inspections as above.

### Composition roles and responsibilities of: the co-ordinating centres, trial management group (TMG) and TSC and data analysis team

Management of the trial is joint between the Centre for Health Sciences Research, University of Salford (Hammond, Ching and Parker) and Lancashire CTU (Forshaw). The TMG (consisting of the authors and CTU trial staff) will approve documentation, study protocol procedures, advise on ethics application, monitor trial progress by reviewing the trial progress reports, any problems arising, be advised of any Serious Adverse Events, review findings and plan dissemination. The TMG will meet at three to four monthly intervals and receive reports of trial progress. Teleconferences and ad-hoc meetings will be held during the study if issues arise requiring discussion.

The TSC consists of both independent and non-independent members (approved by Versus Arthritis). The TSC will meet four times, act as the Data Monitoring Committee (DMC) and provide trial oversight and data monitoring. The TSC will meet to approve the protocol, advice on procedures and progress, data monitoring and review findings and monitor trial progress by reviewing the trial progress reports. No interim analyses are planned.

### Protocol amendments

Any subsequent modifications to the protocol which may impact on the conduct of the study, potential benefit to the patient or may affect patient safety, will require a formal protocol amendment, agreed by TMG and TSC members, and approved by the approving NRES Ethics Committee prior to implementation and notification to sites.

### Dissemination and author eligibility

The full trial protocol will be available via the University of Salford Institutional Repository on trial completion. Topics suggested for presentation or publication will be circulated to the TMG. Lead authorship and author eligibility for articles will be agreed by the TMG. Findings will be submitted to rheumatology, rehabilitation and trial methodology conferences and journals, as relevant. A summary of findings will be provided to relevant health professional and arthritis patient organizations, requesting these are included in websites and newsletters. We will provide a summary of the findings to trial participants via a newsletter and the trial website. If successful, we will also provide training materials and information resources for therapists in delivery of WORKWELL and disseminate these as above and will make these available online.

### Studies within a trial (SWAT)

In addition to the main WORKWELL trial, one SWAT is being conducted.

#### Pre-notification letter/email sub-study

This SWAT will use a randomised controlled trial, embedded within WORKWELL, to test a pre-notification communication sent 2 weeks before participants are due to be sent their 6-month follow-up questionnaire against a control of no pre-notification. The communication will take the form of a letter for participants who opt to complete questionnaires postally and an email for those who opt to complete electronically. The text in the pre-notification communication has been theory-informed. The reminder letter (or email) will be personalised to include the (typed) name of the participant because there is some evidence that personalising may improve response rates in surveys. Randomisation will be stratified by WORKWELL trial arm (WORKWELL intervention; control) and planned method of questionnaire completion (hard copy; online).

The primary outcome is a valid response (i.e. usable outcome data) for the WLQ-25 total score obtained by any means, no more than 56 days after the scheduled 6-month follow-up time-point. The secondary outcomes are: valid response for WORKWELL trial primary outcome (yes/no) without reminder; number of reminders sent; time to response [or ceasing follow-up] (days); costs per participant retained. As is usual with a SWAT, no formal power calculation was undertaken, as the sample size will be constrained by the number of participants sent a 6-month questionnaire. Binary data will be analysed using logistic regression and time-to-response by a Cox proportional hazards model. All models will adjust for stratification factors. We will present a crude analysis of the ratio of the estimated between-groups difference in costs, divided by the corresponding difference in proportions providing valid responses for the WLQ-25.

## Discussion

This protocol describes a definitive, pragmatic, multi-centre superiority randomised parallel group trial, which aims to determine the effectiveness and cost-effectiveness of job retention vocational rehabilitation for employed people with inflammatory arthritis. The results of this trial will inform rheumatology teams, NHS managers and service commissioners about how to optimise JRVR for people with inflammatory arthritis, and about the resources needed to achieve this and considerations for informing its uptake and use in clinical practice (implementation). We will develop online resources for health professionals to support delivery of WORKWELL JRVR, if successful. These resources will be available to support education of future health professionals and be modified to be available for use by people with arthritis. The results will be published in a variety of media with different target audiences, when available.

## Supplementary information

**Additional file 1.** WORKWELL Trial: contents of the: Work Self-help Information Pack; WORKWELL Job Retention Vocational Rehabilitation intervention; Participant Information Sheet and Consent Form.

## Data Availability

The de-identified datasets generated and/or analysed during the current study will not be publicly available (as specific consent to place data in a public database was not obtained) but will be available from the corresponding author on reasonable request. The UK WES-RC and UK WES-RC Manual are available in the University of Salford Institutional Repository (see reference list). The Workwell Solutions Manual will be freely available online at www.workwelluk.org and www.mskhub.comon completion of the trial.

## Abbreviations

AtW: Access to Work
BQ: Baseline questionnaire
CEA: Cost effectiveness analysis
CDF: Contact details form
CF: Consent form
CTU: Clinical Trials Unit
EQ-5D-5L: EuroQol 5 Dimension 5 Level
GCP: Good Clinical Practice
ICER: Incremental Cost Effectiveness Ratio
ICH: International Council for Harmonisation of Technical Requirements for Pharmaceuticals for Human Use
JRVR: Job retention vocational rehabilitation
NHS: National Health Service
NICE: National Institute for Health and Care Excellence
NRS: Numeric rating scale
OMERACT: Outcome Measures in Rheumatology
OT: Occupational therapist
PSN: Participant Screening Number
PIN: Participant Identification Number
PI: Principal Investigator
PsA: Psoriatic arthritis
PT: Physiotherapist
QALYs: Quality Adjusted Life Years
RF: Research facilitator
RA: Rheumatoid arthritis
Rad: Research Administrator
RADAI-5: RA Disease Activity Index-5
RA-WIS: RA-Work Instability Scale
RAID: Rheumatoid Arthritis Impact of Disease scale
RCT: Randomised controlled trial
REDCap: Research Electronic Data Capture
SWAT: Studies Within A Trial
SD: Standard deviation
SPIRIT: Standard Protocol Items: Recommendations for Interventional Trials
SF-12v2: Medical Outcome Survey Short-Form 12 version 2
TM: Trial manager
TMG: Trial Management Group
TRF: Trial registration form
TSC: Trial Steering Committee
UCLAN: University of Central Lancashire
UIA: Undifferentiated inflammatory arthritis
UK: United Kingdom
USA: United States of America
WALS: Work Activities Limitations Questionnaire
WES-RC: Work Environment Survey- Rheumatic Conditions
WLQ-25: Work Limitations Questionnaire-25
WPAI: Work Productivity and Activity Index
WTI: Work Transitions Index
