# Supplementary material for: The effect on work presenteeism of job retention vocational rehabilitation compared to a written self-help work advice pack for employed people with inflammatory arthritis: protocol for a multi-centre randomised controlled trial (the WORKWELL trial)
Source: BMC Musculoskelet Disord. 2020 Sep 10;21:607. doi: 10.1186/s12891-020-03619-1 (PMC7488441; doi:10.1186/s12891-020-03619-1)
Supplement: Supplementary file 1 — Additional file 1. WORKWELL Trial: contents of the: Work Self-help Information Pack; WORKWELL Job Retention Vocational Rehabilitation intervention; Participant Information Sheet and Consent Form. [file 12891_2020_3619_MOESM1_ESM.pdf]

Hammond A et al The effects of job retention vocational rehabilitation compared to a written self-help work advice pack for employed people with inflammatory arthritis: a study protocol for a multi-centre randomised controlled trial (the WORKWELL trial). BMC Musculoskeletal Disorders 2020;.

WORKWELL

KEEPING PEOPLE WORKING

<u>WORKWELL Trial Protocol: Additional File 1</u>

1. Work Self-help Information Pack

This will be received by all participants. The control group only receive this.

During the feasibility study, the participating occupational therapists, patient research partner, working members of the Derby National Rheumatoid Arthritis Society branch (acting as study advisors), and the Trial Management Group, agreed what written information could be representative of “usual care: in terms of work advice provided to people with RA, UIA and PsA. This was further reviewed in preparation for the WORKWELL trial and the range of information booklets increased:

- a welcome letter, encouraging people to read the enclosed booklets and discuss work problems with relatives, friends and employers;
- a self-help flowchart, suggesting how to identify problems, resulting work difficulties and solutions to then implement and/or discuss with employers/ line managers;
- and four work self-help booklets include: a summary of the Equality Act [UK Government Equalities Office, 2010], what “reasonable adjustments” employers could make, common work problems and solutions, the importance of discussing work issues with employers and colleagues and how to get further help [“I want to Work” National Rheumatoid Arthritis

Society, 2018; "Working with Arthritis" Arthritis Care 2016/Versus Arthritis; "Work and Your Wellbeing" Versus Arthritis 2018]..

2. WORKWELL: Job Retention Vocational rehabilitation (intervention group)

WORKWELL is based on that provided in the WORK-IA trial [Hammond et al, 2017] based on the trial conducted by Allaire e et al [2003]..

At the first meeting, the therapist uses the WES-RC with the participant [Hammond et al, 2013 a and b] to:

- Identify work problems and specific barriers (physical, psychological, environmental (physical/social) and managerial) to overcoming these.
- Collaboratively agree three broad priority areas of work problems (e.g. workstation modification and work positioning, moving to/around the workplace), specifying problems within these

The therapist then recommends applicable initial self-management advice tailored to the participant's needs, e.g. hand exercises, relaxation, sleep hygiene, fatigue and/or joint protection advice and provided appropriate booklets, e.g. Versus Arthritis* "Keep Moving" "Fatigue and Arthritis" and "Looking After Your Joints when you have arthritis," as applicable. The participant completes an Action Plan, with the support of the therapist, with a few initial, achievable goals, to meet their identified needs, to help the participant start taking appropriate actions to resolve work problems.

Participants are asked, before the next meeting, to complete an Activity Diary, for 24 hours on a typical work day, identifying for each 30-minute period: their main activity; any equipment, tools or materials used; any difficulties or discomfort doing the activity; whether they took a short rest; and to rate their level of pain and fatigue using a 0 (no) to 10 (severe) scale [modified with permission. This was modified with permission for the UK from an Activity Diary developed by Backman C, Village L, Lacaille D, 2008. Participants were asked to reflect on problems identified in the WES-RC and activity diary and start considering possible solutions. Between the first and second meeting, the therapist reviews the three priority problem areas identified in the WES-RC and develops a range of possible solutions to discuss/ practice with the participant.

At subsequent meetings, the therapist and participant reviewed progress with action plans set in the previous meeting, collaboratively problem-solve, and the therapist continues supporting participants to write Action Plans and in resolving difficulties for themselves. Throughout the meetings, therapists emphasise the importance of participants taking responsibility to liaise with employers to request and obtain job accommodations.

WORKWELL is individualised to include (based on the WES-RC, activity diary and priority problem areas), as applicable:

a. applying ergonomic, fatigue and stress management approaches to the workplace
b. recommendations for assistive technology/equipment adaptation, workplace/workstation modification, transport advice
c. practical advice and support enabling participants to disclose their condition and negotiate job modifications with employers, e.g. discussion of strategies, how to explain their condition its impact on work, proactively make suggestions for addressing issues and role play, when applicable.
d. explaining rights under the Equality Act 2010, how and why their condition meets being one applicable under the Equality Act, what are "reasonable adjustments" employers can make in the workplace, and the facilities available to support employers making "reasonable adjustments," e.g. Access to Work [https://www.gov.uk/access-to-work]; and how the participant could apply to Access to Work.
e. psychological support, through listening to and discussing work problems; encouraging ability and confidence in solving work problems, managing arthritis when working and continuing working in future
f. advice on other activities of daily living and hand function difficulties affecting work ability
g. to discuss, if necessary, about considering a change in job or career. Referral to a Disability Employment Advisor (DEA) at their local Jobcentre for advice and support in doing so, if the participant wishes
h. referral to other relevant services, such as 'mainstream' occupational therapy (e.g. for splinting, activities of daily living training, self-management education), physiotherapy (e.g. for pain management, exercise therapy), podiatry (e.g. for insole or footwear provision), or other work rehabilitation services (e.g. DEA; advice to contact their employer's occupational health department; contact with Access to Work).

i. provision of relevant work and self-management advice booklets and other information as appropriate
j. general advice on disease flare management and contacting the Rheumatology department quickly in a flare, or problem joints, for review of medication changes or steroid injection, if necessary, to reduce the risk of longer sickness absences
k. optional: if applicable, to conduct a worksite visit to conduct a workplace job assessment and employer liaison. A meeting with the therapist, participant and their line manager together to discuss job accommodations that can be made; and enable referral to Access to Work for equipment / other support funding, as applicable. The content of this meeting will be discussed with the participant in advance. The participant provides written consent to the work site visit and obtains permission in advance from the employer for this to occur. The therapist, as necessary, liaises in advance with the workplace to ensure any health and safety issues attending the worksite are explained and appropriate arrangements made. Alternately, employer liaison may be by telephone and with the participant's involvement (with agreement about the content of discussion in advance).
l. A telephone review, six weeks after the final meeting, to discuss participants' progress implementing changes, check recommended job accommodations ae in place and if any further action was needed.
m. The participant is provided with a discharge letter, summarising the issues, actions and recommendations provided in WORKWELL. This is written to be suitable to provide to the employer/ line manager, if the participant chooses to do so.

*Versus Arthritis downloads available from:

Keep Moving:

https://www.versusarthritis.org/media/1310/keep-moving-information-booklet-with-poster.pdf

Looking After Your Joints:

https://www.versusarthritis.org/media/1271/looking-after-your-joints-information-booklet.pdf

Fatigue and Arthritis:

https://www.versusarthritis.org/media/1269/fatigue-and-arthritis-information-booklet.pdf

3. <u>Participant documents</u>

PARTICIPANT INFORMATION SHEET:

WORKWELL: Testing work advice for people with arthritis

<u>Part One: Introduction</u>

We would like to invite you to take part in a research study. The study is being run by the Centre for Health Sciences Research, University of Salford. The Lancashire Clinical Trials Unit, University of Central Lancashire, is also supporting the trial. Before you decide, you need to understand why the research is being done and what it will involve for you. Please take time to read the following information carefully. Talk to others about the study if you wish. The research nurse/ facilitator at your hospital will be happy to go through this information sheet with you. They can answer any questions you have. Alternately, our **Trial Research staff (XXXX)** can also do this on the telephone. Please ask if there is anything that is not clear to you or if you would like more information. Take time to decide whether or not you wish to take part.

What is the purpose of this study?

Working people with inflammatory arthritis (i.e. rheumatoid arthritis, early inflammatory arthritis or psoriatic arthritis) may find that they have some difficulties doing their job because of their arthritis. Work issues might be: physical, managing the effects of arthritis at work, psychological; relationships with co-workers or managers; and/or balancing work, health and personal life.

The advice and help available for working people with arthritis about work issues varies considerably between Rheumatology departments and also between people's workplaces. In this study we want to **test two ways** of providing working people with arthritis with advice and support about how to manage any work problems they may have. These are for people who have concerns about their health affecting their ability to work over the next few years.

We are testing:

a) A work self-help information pack: Information booklets, specifically designed for working people with arthritis, with practical advice about work issues and how to manage these. **Plus**

written guidance on identifying your work issues, where you can get support and any actions or changes you can make yourself, if you wish to.

b) The self-help information pack **plus** practical advice from a therapist at your Rheumatology department (i.e. the WORKWELL programme). The therapist will offer between 2 to 4 appointments of about 45 to 60 minutes each. These will usually be in the occupational or physiotherapy department at your hospital. The number of appointments depends on your work-related needs. These are usually about a month apart and flexibly timed around your work commitments (for example, appointments can be earlier or later in the day). The therapist will work with you to identify your work issues, discuss possible practical solutions and help you with any changes you decide on.

People taking part are allocated by chance (by a computer) to one or other of these two types of work advice. We are asking about 240 working people with inflammatory, rheumatoid or psoriatic arthritis to take part. You are asked to fill out a questionnaire booklet before receiving one of the two types of work advice. We will then ask you to fill out a questionnaire booklet 6 months later and again 12 months later. Each month, we will also ask if you took any days off due to ill-health. You would therefore be taking part in the study for about a year.

There is little research in the UK about whether work advice helps people with arthritis to reduce their work problems or stay in work. Two studies in the USA have shown work advice can help people stay in work for longer, but two studies in the Netherlands showed it made little difference. However, the USA and the Netherlands are very different to the UK. We want to see if this work advice helps working people with arthritis in the UK and whether it is value for money to provide this in NHS Rheumatology departments. We are asking for your help with this study.

Why have I been invited?

Your Consultant Rheumatologist, Rheumatology nurse, occupational therapist or physiotherapist helped identify this study might be relevant for you. We are asking people to take part who:

1. Have **one** of three types of arthritis:

- Early (undifferentiated) inflammatory arthritis (EIA); Rheumatoid arthritis (RA) or Psoriatic arthritis (PsA).

2. Are employed (including self-employed) for 15 hours or more (on average) per week.

3. Are experiencing some work difficulties due to arthritis.

4. Are **not on sick leave**. BUT if you are on short-term sick leave (that is, due back at work soon and you won't be off work for more than 4 weeks altogether), you can still take part. You will need to have been back at work for at least a few days first. This is because some questions in the questionnaire booklet ask how you have been managing <u>at work</u> "<u>within</u> the last 2 weeks" If you are interested in taking part but on sick leave, discuss this with the research nurse / therapist at your hospital who has given you this information sheet. You can also contact the researchers at the University of Salford (see contact details at end of this information sheet). We can discuss with you about joining the study when you are back at work, if you wish.

5. Are **not** planning to retire or take early retirement (through choice or ill-health) in the next 12 months

6. Are **not** planning to move out of the Rheumatology department catchment area within the next 3 months. (That is, you can attend the hospital if you were asked to come to the practical advice sessions with a therapist).

7. Are **not** already receiving or recently received work advice from work rehabilitation services, such as Access to Work. If you have received / are receiving advice from your occupational health or human resources department at work, you **can** still take part.

Do I have to take part?

It is up to you to decide. Take your time to do so. Please discuss it further and ask questions with the person giving you this information. If you want to ask anything further, please call us (**WORKWELL Trial Research Staff)** and we will be happy to answer any questions. (You can also send us the Contact Details Form in the FREEPOST envelope enclosed, if you are interested and want more information. We can then call you).

If you are interested in taking part, the research nurse or therapist providing you with this information will explain further about the study and first check if you are eligible to take part (i.e. trial screening). We will be using the fully anonymized information from this screening to summarize, for example, the ages, gender and types of jobs of people who express an interest in taking part.

If you decide not to take part, this will not affect the standard of care you receive. If you decide to take part, you can still withdraw at any time. You do not need to give a reason. This will not affect the standard of care you receive.

What will happen to me if I take part?

1. **If you decide to take part:** we will ask you to sign a consent form to show you agreed. The research nurse or therapist at your hospital will complete the consent form with you. With your consent, your Consultant Rheumatologist will be informed that you are taking part in this study. Following your consent, the research nurse/ therapist at your hospital will provide your NHS Number to add to your "Trial Registration Form" (i.e. the form confirming to the WORKWELL trial research staff that you agree to take part in the trial). This enables accurate identification of you **if** there is a need for the researchers to contact your own health care team in future. This would be because : the researchers have unexpectedly been unable to contact you for a long period; to help us contact you again in 4 years-time to ask how you are getting on (see point 11); or there are health-related concerns requiring reporting as part of the researchers' duty of care to you.

If you cannot come to the hospital to complete the trial screening and/ or consent form: you can complete trial screening and /or consent by telephone and mail with the WORKWELL Trial staff. <u>We will need your contact details to do this</u>. The research nurse or therapist, discussing the study with you, will **only** send your contact details to us **if** you agree to this. Or please mail your contact details to us yourself on the Contact Details Form enclosed, using the FREEPOST envelope. We can then call you to discuss the study, check to see that you are eligible and answer any questions you may have before you decide to complete your consent.

<u>On the Contact Details Form:</u> please provide both your land-line and <u>mobile</u> telephone numbers (if you have them); and your <u>e-mail address</u> (if you have one).

- **Let us know** the best time/days to contact you. **If we can** call/text/e-mail you at any reasonable time of the day/ early evening: state "anytime."

This will make it much easier for us to get in touch with you. We can leave text/ voicemail/ e-mail messages to arrange talking to you. We can call you early evenings if you are out at work all day.

If you decide to take part, we may also use your contact details later if we need to get in touch about your study questionnaires (for example, if you missed out anything by mistake). If you are referred to the WORKWELL programme, we will also let the therapist have your contact details so they can make an appointment with you. Knowing your day-time availability, or that messages can be left on your mobile/ e-mail, will make it much easier for them to get in touch with you. The therapists usually don't work in the evenings.

2. Following consent, we will give/mail you a **questionnaire** to complete to do in your own time. If you prefer to do this **online**, we can e-mail you a link to a secure online questionnaire, only accessible to you and the research team. This questionnaire will ask about, for example, your age, diagnosis and medication. It will also ask about your work, what work difficulties you have, how your health is affecting your work, your work-life balance and other health-related information. The questions are mostly answered using tick boxes/ circling numbers. It takes about 45-60 minutes to fill in. You do this in your own time at home (paper or online). Then you post it back to us in the FREEPOST envelope provided (i.e. at no cost to yourself) or just submit the online version. (The online version is only available for the 6- and 12-month follow-up questionnaires).

3. Within one week of returning the questionnaire, you will receive the work self-help advice pack through the post from the Lancashire Clinical Trials Unit. A letter with this will let you know whether or not you will also receive the WORKWELL programme.

4. If it was decided by chance that you receive the self-help information advice pack to use on your own, we ask that you read the guidance and information. Please identify the work problems you are experiencing and who you could discuss these with (e.g. family, friends, employer, GP). Take time to talk to people and think about solutions. Write down your own Action Plan. Then make any changes you decide will help.

5. If it was decided by chance that you will receive the pack plus the WORKWELL programme, within 1 to 2 weeks a therapist will get in touch to make the first appointment with you. This will be at the occupational therapy or physiotherapy department at the hospital you normally attend. When you are referred, we will send the therapist your contact details to help the therapist contact you. They can offer appointments earlier or later in the day to reduce the impact on your working day.

In the first meeting, the therapist helps you identify your work problems. In the following meeting(s), the therapist discusses and agrees with you any changes you want to make and gives practical advice about how to make changes. Usually the WORKWELL programme lasts 2 to 4 meetings. Each can be up to one hour, and each are about a month apart. The therapist will call you a few weeks later to ask how you are getting on. **Sometimes,** the therapist **might** suggest a work site visit would also be helpful to see what your problems are. **If** this would be helpful, the therapist will discuss this with you. It is up to you if you want this to happen or not. If yes, the therapist would need your line manager/ employer's permission. We understand that not everyone has told their employer/ people at work that they have a health condition.

- At the end of the WORKWELL programme, the therapist will write a report for you about the main work issues identified and advice given. You can use this to remind yourself about what you have done/ can do. You may find it helpful to discuss this with your line manager/ employer. It is up to you.
- **Optional**: we would like to audio-record the first appointment that your Workwell therapist has for one patient. You **might** be asked if the therapist can record your first appointment. This is so we can later listen to how the therapist provides treatment; and we will take notes about what is said. You can let us know if you agree to this on the consent form. Not everyone will be asked by the therapist. You can still change your mind at any time. The audio-recordings will be transferred securely to the research team. The therapist then deletes their copy. Recordings will only be accessed by our research team. Your name is not identified in any notes or reports. We delete the recordings afterwards.

6. Six months after you complete the first questionnaire, we will mail you a second one/ send a link to the online questionnaire (whichever you prefer). This is shorter and asks about: work-related problems, health-related information and what you thought about the type of work advice you

received. This takes about 30 minutes. You do this in your own time at home. Then you post it back to us in the FREEPOST envelope provided.

7. Another six months later (12 months from the start), we ask you to complete the third questionnaire. This is also about: any work difficulties you may have, how your health may have affected your work, work-life balance, other health-related information, and any other treatment you had in the last 6 months. This takes about 45 to 60 minutes. You do this in your own time at home. Then you post it back to us in the FREEPOST envelope provided/ submit the online questionnaire (whichever you prefer).

8. **Each month for the 12 months: we will ask if you have been off work because of ill-health that month, and if yes, how many days it was.** We can do this by text, e-mail or phone, as you prefer.

After the final questionnaire (at 12 months):

9. **Optional:** we would like to interview about **15 people who received the WORKWELL programme (i.e. just over 10%)**. You can let us know if you are interested on the consent form. If you prefer not to be interviewed, it's not a problem. We don't need everyone to help. If you do agree to the interview, you can still change your mind at any time. In the interview, we ask your views about the work advice you received. The interview may be by telephone or in person and at a time and place that suits you (e.g. your home or hospital you usually go to) and will last up to 45 minutes. We would like to audio-record it to help us later in writing down accurately what you said. The recordings are only listened to by our research staff. Your name is not identified when these are typed up. We delete the recordings once the content is written down. We will not ask about any private information. (Please do not disclose such information to the interviewer).

10. **Optional:** we will write to some people who attended the WORKWELL programme to ask if they are interested in us interviewing their employer/ line manager about what their views of the work advice you received and if they think there are any benefits for the company/ organization you work for. This is a 15-20-minute telephone interview. We **only** need about **10%** of people to help us contact their employer/ line manager. Therefore, if you don't want to do this, it's not a problem. It does not

affect you taking part in the study now in any way. **If** in a year's time you are interested, we will give you more information, you can think about it and take time to decide. **If** you are interested, we ask if you will talk to your line manager/ employer about being interviewed, and to help put us in touch if they are. We send them information to think about and they can decide then.

11. **Lastly,** we would like to see how you get on in the future with your working life. We would like to send you another copy of the questionnaire in **three years' time from now**. You can decide then if you want to complete this.

We will send you a thank you letter when you have finished the third questionnaire (at 12 months). We will ask you, in the thankyou letter, if you would be interested in letting us know about what you thought about taking part in the trial and your ideas about how the treatments, we are testing could best be put into practice when we know the results. You can decide then if you want to do this. Our "Workwell Patient Group" is leading this.

We will also let you know the results of the study when it has ended (which will be in 2022).

Expenses and payments

There is no expense in completing the questionnaires, as we provide FREEPOST envelopes for the reply. We will pay back any travel costs you have attending clinics or therapy departments to complete consent, for the WORKWELL programme or for an interview. We provide a claim form. Please attach any receipts (for bus, train, parking) to this. Include the number of miles travelled if you come by car.

As a "thank you," on returning each of questionnaires we send you at 6 and 12 months, we will enter you into a **Prize Draw** at the end of the study (late 2021/ early 2022). Returning both questionnaires will mean you have two Prize Draw entries. There are five prizes worth the equivalent of **£50** each. Please let us know if you would like to be entered or not (by answering this question at the end of the first questionnaire).

What are the possible benefits of taking part?

The study may help you, if you choose to make changes suggested in the work information pack or WORKWELL programme, although it may not help. The information you give us will help us understand

any effects the information pack and WORKWELL programme may have on people with arthritis' work problems, and if one method of providing work advice is better than the other. In future, this will help therapists plan work-related treatment for people with arthritis. It can help the NHS plan appropriate work-related services for people with arthritis.

What are the possible disadvantages and risks of taking part?

The work advice pack contains information available to people with arthritis, if they know where to find it. The WORKWELL programme is provided by a therapist at your hospital who has received training in this. We do not expect there are any disadvantages or risks to you in taking part, as you choose what changes (if any) to make. If you choose to take part in the interview, we will arrange this at a place and time to suit you.

If the information in Part 1 has interested you and you are considering taking part, please read the additional information in Part 2 before making any decision.

Part Two: Further information

Will my taking part in this study be kept confidential?

Yes. We will follow ethical and legal practice. We will handle all information about you in confidence. We will store it securely at the University of Salford and the Lancashire Clinical Trials Unit. All information which is collected about you during the course of the research will be kept strictly confidential. We only identify you in the interview recording by a number. You have the right to check the accuracy of the data held and correct any errors. Only staff involved in the research study see your information. Any direct quotes from the audio-recordings we use in reports, will not identify you in any way. We send a copy of your consent form to the hospital Consultant responsible for your care. This will be placed in your medical records.

How we will use your personal data and your rights under the law.

The University of Salford is the sponsor for this study based in the United Kingdom. We will be using information from you in order to undertake this study and will act as the data

controller for this study. This means that we are responsible for looking after your information and using it properly.

Your rights to access, change or move your information are limited, as we need to manage your information in specific ways in order for the research to be reliable and accurate. If you withdraw from the study, we will keep the information about you that we have already obtained. To safeguard your rights, we will use the minimum personally-identifiable information possible.

You can find out more about how we use your information by contacting **Prof Alison Hammond and on our website:** **https://www.workwelluk.org/privacy-notice**

The NHS department you attend which introduces you to this study, will collect information from you and your medical records for this research study in accordance with our instructions. The department will use your name and contact details to contact you about the research study, and make sure that relevant information about the study is recorded for your care, and to oversee the quality of the study. Individuals from the University of Salford and regulatory organisations may look at your medical and research records to check the accuracy of the research study. Your NHS department will pass these contact details to us at the University of Salford, along with the information collected from you and your medical records. The only people in the University of Salford and the Lancashire Clinical Trials Unit who will have access to information that identifies you will be people who need to contact you to (for example to discuss the study, find out any missing information, arrange an interview) or audit the data collection process. The people who analyse the information will not be able to identify you and will not be able to find out your name or contact details.

Your NHS department will keep identifiable information about you from this study for 3 years after the study has finished. The research team at the University of Salford will also keep identifiable information about you from this study for 3 years after the study has finished. Your information will be stored in locked cabinets and then a data archive store within the Centre for Health Sciences Research and only accessible to members of the research team. After this date, your identifiable information, questionnaires (paper and any scanned information) will be confidentially destroyed or purged from the University server.

What will happen if I don't want to carry on with the study?

You can withdraw from the study at any time, and your care will not be affected. We will use the data collected up until your withdrawal unless you tell us not to.

What will happen if there is a problem?

If you have problems with the WORKWELL programme, please contact and discuss this with the therapist who you saw. If you have any queries or problems about the work advice pack, the study, questionnaires or interview, please contact our WORKWELL Research Staff who will do their best to help.

If you have any complaint about the way you have been dealt with during the study, or any possible harm you might suffer, we will address this. If you have a concern about any aspect of this study, you should ask to speak first to: the researchers and, if appropriate, the therapist who provided the WORKWELL programme to you. We will do our best to answer your questions (contact our WORKWELL Research Staff – see below).

If you remain unhappy and wish to complain formally, you can do this through the School of Health Sciences, University of Salford. Please contact:

Chair of the Health Research Ethics Panel, Frederick Road Campus, University of Salford, Salford, M6 6PU.

Tel: xxx. Email: xxxx

If you remain unhappy and wish to complain formally through the NHS Complaints Procedure, details can be obtained from:

The Customer Care Manager

Address of the participating hospital

Tel: xxxx

In the event that something does go wrong and you are harmed during the research, and this is due to someone's negligence, then you may have grounds for a legal action for compensation against the NHS Trust at which you received the work advice and/or the University of Salford, but you may have

to pay your legal costs. The normal National Health Service complaints mechanisms will still be available to you (if appropriate).

What will happen to the results of the research study?

We will send you a summary of the findings when the study is fully completed. When you finish taking part, we will send you a letter saying when this is likely to be.

We will write up the findings and submit them for publication. This will help Rheumatology and Therapy departments learn from our experiences. You will not be identified in any report or publication. We will send your therapist and their Rheumatology/ Therapy department a summary of the study when it is finished. We will also present the study results at national and international rheumatology conferences. We will inform arthritis charities so they can choose whether to make the findings available to other people with arthritis via their websites/ magazines.

Who is organizing and funding the research?

The Centre for Health Sciences Research, University of Salford and the Lancashire Clinical Trials Unit are organizing this trial. We are also working with researchers at the: University of Manchester, University of Lancaster, University of Southampton, and the Derby Teaching Hospitals NHS Foundation Trust.

The study is funded by Versus Arthritis (formerly Arthritis Research UK). The Chief Investigator is Professor Alison Hammond. The Centre for Health Sciences Research, University of Salford is part of the Arthritis Research UK/ Medical Research Council's national Centre for Musculoskeletal Health and Work Research.

Who has reviewed the study?

All research in the NHS is looked at by independent groups of people, called a Research Ethics Committee, to protect your safety, rights, wellbeing and dignity. This study has been reviewed and given favourable opinion by West Midlands-Solihull Research Ethics Committee. The Research Ethics Committee at the University of Salford and University of Central Lancashire have also approved the study.

Further information and contact details

If you have any questions about the study, wish to discuss taking part or have any concerns; please contact either:

At your hospital: *[Optional: some sites may wish to have their research nurse/ lead therapist contact details entered here]*

Or you can contact the researchers direct at:

Trial Manager: **Trial Research Administrador:**

Chief Investigator: Prof. Alison Hammond

Centre for Health Sciences Research, University of Salford, Allerton, Frederick Road, Salford M6 6PU

Information about the study is also available from our website: www.workwelluk.org

If you have any general queries about participating in research you can contact your hospital's Patient Advisory and Liaison Service (PALS). Your PALS can be contacted at:

XXXXXXXXXXXXXXXX

General information is also available on the INVOLVE website (www.invo.org.uk). **INVOLVE** is a national advisory Group, funded by the Department of Health, which supports active public involvement in NHS, public health and social care research.

Finally

Thank you for considering taking part. Please keep this information sheet. We will ask you to sign a consent form if you agree to take part. We will give you a copy of this to keep.

Patient Screening Number:

S						

WORKWELL CONSENT FORM

Title of project: WORKWELL: Testing work advice for people with arthritis

Name of researcher: Prof Alison Hammond **Please INITIAL all boxes (i.e. do NOT tick)**

1. I confirm that I have read and understand the information sheet dated 9.9.19 **(Version 3**) for the above study. I have had the opportunity to consider the information, ask questions and have had these answered satisfactorily.
2. I understand that my participation is voluntary and that I am free to withdraw at any time, without giving any reason, without my medical care or legal rights being affected.
3. If I do later choose to withdraw from the study, I agree that any data collected up to that point can be kept and used in the study, unless I inform the researchers otherwise.
4. I agree to participate and understand that I will receive a work self-help information pack and I may also be allocated to attend the WORKWELL programme at my Rheumatology/ Therapy department.
5. **Optional:** If I see a therapist as part of the research: I agree to allow one appointment to be audiorecorded by the therapist. I understand that: this will be securely sent to the research team; the therapist will delete their copy; the recording is deleted once transcribed; and anonymised quotes may be given verbatim in reports.
6. **Optional:** if I see a therapist as part of the research: I agree to take part in the face-to-face interview about the work advice I receive. I understand that the interview will be audio-recorded, recordings will be deleted once transcribed and anonymised quotes may be given verbatim in reports.
7. I understand that relevant sections of my medical /therapy notes may be looked at by members of the research team, regulatory authorities or from the NHS Trust, where it is

relevant for my taking part in this research. I give permission for these individuals to access my records.

8. I understand that my personal details will be kept confidential and will not be revealed to people outside the research team
9. I agree to my Rheumatology Consultant being informed of my participation in this study.
10. I understand a copy of this form and my contact details will be forwarded by the Research Facilitator/ therapy team at my hospital to the research team at the University of Salford and to the Lancashire Clinical Trials Unit.
11. I understand that my fully anonymised data will be used in research presentations, reports and articles.
12. I agree to take part in the above study.
13. I agree to being contacted in future to **ask about** taking part in a longer-term follow-up for this study and other associated studies. I understand that I can change my mind about this at a later date.

Name of patient:_____________________Date:____________**Signature:**_________________

Name of person
taking consent:______________________ Date:____________ **Signature**:________________

When completed copy x3: 1 for patient; 1 for medical notes; 1 for WORKWELL Trial Manager (UoS); and file original in WORKWELL site file.
